# Loss of spermatogonia and wide-spread DNA methylation defects in newborn male mice deficient in DNMT3L

**DOI:** 10.1186/1471-213X-7-104

**Published:** 2007-09-18

**Authors:** Sophie La Salle, Christopher C Oakes, Oana R Neaga, Déborah Bourc'his, Timothy H Bestor, Jacquetta M Trasler

**Affiliations:** 1 Departments of Pharmacology & Therapeutics, Pediatrics and Human Genetics, McGill University and The Montreal Children's Hospital Research Institute, Montréal, QC, H3H 1P3, Canada; 2 INSERM U741/Paris 7 University, 75251 Paris Cedex 05, France; 3 Department of Genetics and Development, College of Physicians and Surgeons of Columbia University, New York, NY 10032, USA; 4 The Jackson Laboratory, Bar Harbor, ME 04609, USA

## Abstract

**Background:**

Formation of haploid spermatozoa capable of fertilization requires proper programming of epigenetic information. Exactly how DNMT3L (DNA methyltransferase 3-Like), a postulated regulator of DNA methyltransferase activity, contributes to DNA methylation pattern acquisition during gametogenesis remains unclear. Here we report on the role of DNMT3L in male germ cell development.

**Results:**

A developmental study covering the first 12 days following birth was conducted on a *Dnmt3L *mutant mouse model; lower germ cell numbers and delayed entry into meiosis were observed in *Dnmt3L*^-/- ^males, pointing to a mitotic defect. A temporal expression study showed that expression of *Dnmt3L *is highest in prenatal gonocytes but is also detected and developmentally regulated during spermatogenesis. Using a restriction enzyme qPCR assay (qAMP), DNA methylation analyses were conducted on postnatal primitive type A spermatogonia lacking DNMT3L. Methylation levels along 61 sites across chromosomes 4 and X decreased significantly by approximately 50% compared to the levels observed in *Dnmt3L*^+/+ ^germ cells, suggesting that many loci throughout the genome are marked for methylation by DNMT3L. More so, hypomethylation was more pronounced in regions of lower GC content than in regions of higher GC content.

**Conclusion:**

Taken together, these data suggest that DNMT3L plays a more global role in genomic methylation patterning than previously believed.

## Background

DNA methylation is central to epigenetic control of the genome [1]. In mice, targeted inactivation of DNA methyltransferase genes causes lethality at early embryonic or postnatal stages, emphasizing the importance of DNA methylation in supporting mammalian development [2,3]. Germ cell development is the first developmental window during which DNA methylation patterns are programmed in mammals. Marked differences are observed between male and female gametes, especially at imprinted loci where allele-specific gene expression in the offspring is dependent on methylation differences [4]. The development of a number of human diseases, including Beckwith-Wiedemann, Prader Willi and Angelman syndromes, has been linked to aberrant expression of imprinted genes [5].

In the mouse, a major demethylation event takes place in both germ lines between embryonic days (E) 10.5 to 12.5 as primordial germ cells (PGCs) enter the gonads, following which point DNA methylation patterns are reestablished in a sex- and sequence-specific manner as gametogenesis progresses [6,7]. Most information concerning the dynamics of DNA methylation comes from studies done on the control region of imprinted genes and repeat sequences [8-16]. In the male germ line, methylation acquisition begins before birth, between E15.5 and E18.5. Repetitive elements such as intracisternal A particles (IAPs), long interspersed nuclear elements (LINEs) and satellite sequences have acquired most of their methylation by E17.5 [12,16]. Although the remethylation of imprinted genes also begins around E15.5, the process is only completed after birth [8,9,13]. A developmental study done on the imprinted gene *H19 *shows that it begins acquiring its methylation between E15.5 and E18.5, but only becomes fully methylated postnatally by the pachytene spermatocyte stage [8,9]. Importantly, since male germ cells continue to undergo DNA replication after birth, they are also capable of maintenance methylation to ensure proper propagation of the patterns already acquired. In contrast, in the female germ line methylation patterns are acquired postnatally during the oocyte growth phase, once DNA has been replicated and pachynema is completed [11,14,16].

DNA methylation patterns are created and propagated through the activity of both *de novo *and maintenance DNA (cytosine-5)-methyltransferases (DNMTs). A number of DNMTs have been characterized and are classified according to similarities found in their catalytic domain [17]. However, we are only beginning to understand how these enzymes interact in the germ line to establish and maintain methylation patterns. We have previously shown that the expression of *Dnmt1*, *Dnmt3a*, *Dnmt3b *and *Dnmt3L *marks windows of sex-specific methylation in both germ lines, and more recently that the expression of *Dnmt3a *and *Dnmt3b *is tightly regulated in developing male germ cells [18,19]. Germ cell-specific inactivation of *Dnmt3a*, but not *Dnmt3b*, impairs the establishment of *de novo *methylation patterns in germ cells, more specifically at imprinted loci, without affecting the methylation status of interspersed repeat sequences [20].

Targeted inactivation of the DNA methyltransferase 3-Like gene, *Dnmt3L*, also results in methylation defects at imprinted loci in both germ lines and additionally, in male germ cells, at dispersed repeated sequences such as IAPs and LINEs [20-24]. Spermatogenesis is impaired in *Dnmt3L *mutant males due to abnormal synapses between homologous chromosomes resulting in meiotic failure [22,24]. Although DNMT3L shares common motifs with DNMT3a and DNMT3b, it lacks the ability to transfer methyl groups to DNA. Both cell culture studies and *in vitro *biochemical assays have shown that DNMT3L interacts with and stimulates the *de novo *methylation activity of DNMT3a and its isoform DNMT3a2, as well as DNMT3B [25-29]. Although concomitant expression of *Dnmt3a2 *and *Dnmt3L *in gonocytes has previously been reported [30,31] and *Dnmt3L *mutant mouse models have clearly established the importance of this protein to the germ line [21,23,24], the action of DNMT3L – alone or in combination with another DNMT – is still not fully understood.

Spermatogenesis is a complex process by which haploid male germ cells are created. Sperm integrity not only depends on unique processes such as specialized transcription, meiosis and histone-to-protamine replacement, but also on various epigenetic events including DNA methylation reprogramming [32,33]. Treating male mice with the cytosine analogue 5'-aza-2'-deoxycytidine, an agent that causes genomic hypomethylation, results in testicular abnormalities with decreased sperm counts and fertility [34]. A recent study by Marques *et al*. [35] showed that imprinted gene defects were associated with oligozoospermia in humans, suggesting that proper DNA methylation programming may be required for spermatogenesis to progress normally. Although little is known about the functional requirement(s) of methylation at single-copy or repeat sequences on germ cell proliferation and differentiation, these results suggest a crucial role for DNA methylation in male germ cell development.

Our analysis here of *Dnmt3L *function in the male germ line revealed a more prominent and developmentally earlier role for this protein during spermatogenesis than was previously reported. Using a *Dnmt3L *gene-inactivation mouse model [21], we determine that histological abnormalities were already occurring during the first week of postnatal development. Lower germ cell counts and delayed entry into meiosis were observed in *Dnmt3L*^-/- ^males. A thorough developmental study exploring the expression of *Dnmt3L *in both prenatal and postnatal male germ cells showed that, although *Dnmt3L *expression peaked in gonocytes, it was also detected in spermatogonia, spermatocytes and spermatids. Finally, DNA methylation analysis of chromosomal domains revealed that DNMT3L was crucial to the establishment of global DNA methylation patterns in the male germ line.

## Results

### Timing of appearance of abnormal testicular histology in Dnmt3L^-/- ^males

Three mutant mouse models have been created to study the role of *Dnmt3L *in the germ line [21,23,24]. All studies have reported the appearance of clear histological defects starting at two weeks after birth, including abnormal synapsis of homologous chromosomes and accumulation of ribosomal particles in spermatocytes, as well as decreased proliferation and loss of germ cells by apoptosis [21-24,36]. We were intrigued by the fact that the consequences of depriving germ cells of a protein that is so highly expressed before birth would appear only after cells have resumed mitosis, differentiation, and entered meiotic prophase. In hopes of delineating the timing of the defect, we conducted a detailed histological study of developing *Dnmt3L *mutant testes using the model created by Bestor and colleagues [21]. Testes were collected at two-day intervals, starting from 4 days postpartum (dpp) until 14 dpp, the time at which defects have been reported to appear. Between 2 and 4 dpp, gonocytes or prospermatogonia re-enter mitosis and relocate to the basement membrane. Some germ cells are set aside to become spermatogonial stem cells, while the rest enter the first wave of spermatogenesis, maturing into type A spermatogonia [37]. Between 7 and 8 dpp, the more mature dividing type A spermatogonia differentiate into intermediate and then type B spermatogonia, the last cell type to mitotically divide before entering meiosis. Cells first engage in meiosis between 9 and 10 dpp, as DNA is replicated one last time in absence of cell division [38].

**Figure 1:**
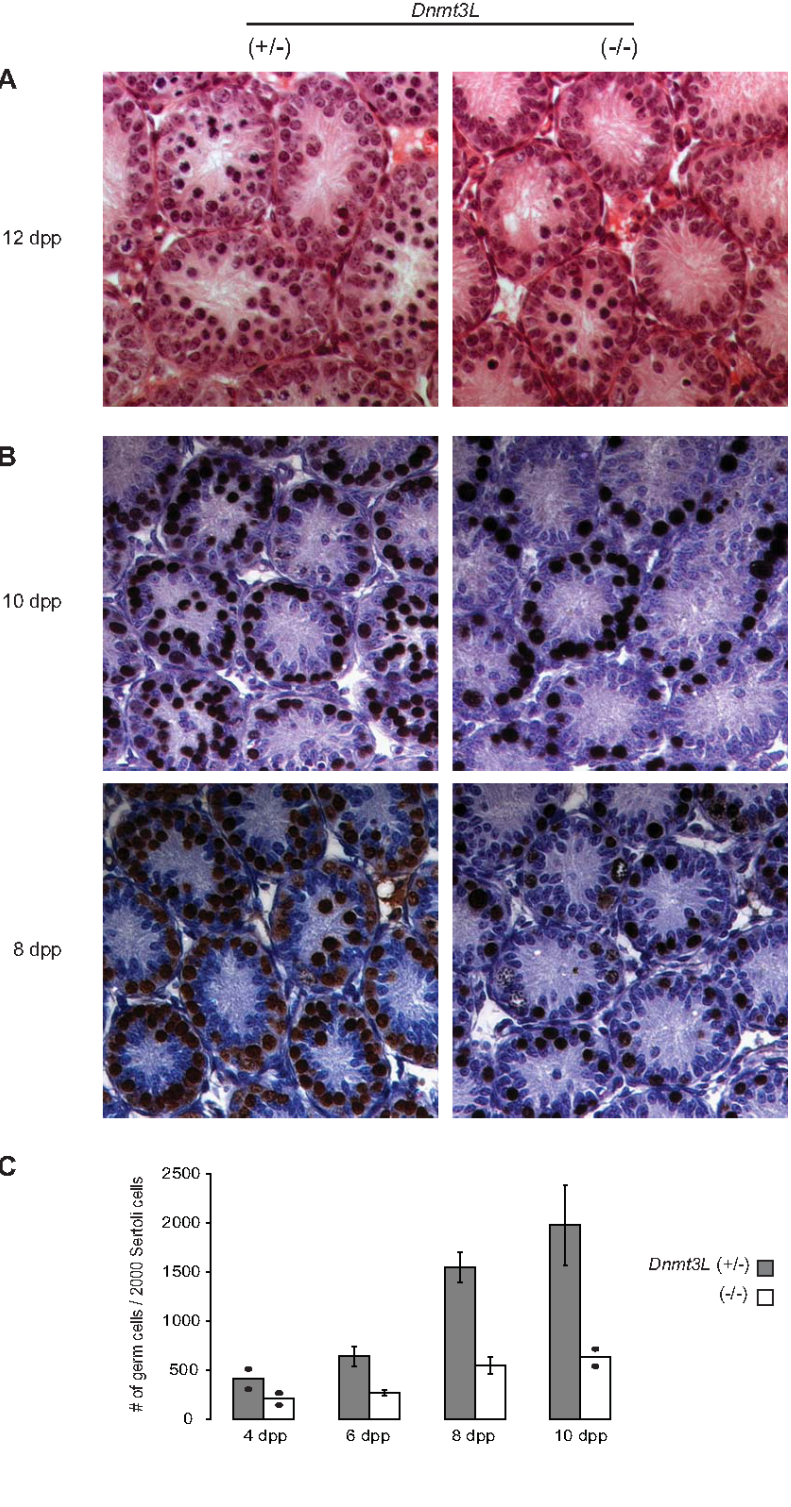
**Histological abnormalities and decreased germ cell counts in newborn *Dnmt3L*^-/- ^testis**. **A) **Hematoxylin and eosin staining of cross-sections of testes from 12 dpp *Dnmt3L*^+/- ^(left) and *Dnmt3L*^-/- ^(right) mice. Germ cells do not appear to be entering meiosis in the absence of DNMT3L (right panel). **B) **Immunoperoxidase staining of germ cells with the GCNA1 antibody (brown) in testis sections of 10 dpp (top) and 8 dpp (bottom) *Dnmt3L*^+/- ^and *Dnmt3L*^-/- ^mice. **C) **Graphical representation of germ cell counts in *Dnmt3L*^+/- ^and *Dnmt3L*^-/- ^testes. A difference in germ cell count is observed as early as 6 dpp in absence of DNMT3L. By 10 dpp, mutant males have approximately a quarter of the number of germ cells their *Dnmt3L*^+/- ^littermates have. For each time point, GCNA1-positive germ cells were counted per 2000 Sertoli cells; two to three males were examined per genotype. Results are presented as means ± SEM; mean counts for individual males are presented as dots when only two animals were analyzed.

Histological examination of *Dnmt3L*^-/- ^testes at the light microscope level revealed no detectable morphological differences at 4, 6 and 8 days postpartum when compared to *Dnmt3L*^+/+ ^and *Dnmt3L*^+/- ^testes (data not shown). Germ cells had relocated to the basal compartment and dividing cells were present in *Dnmt3L*^-/- ^as they were in *Dnmt3L*^+/+ ^and *Dnmt3L*^+/- ^testes. While testicular histology of *Dnmt3L*^+/+ ^and *Dnmt3L*^+/- ^males remained the same at all time points examined, perceptible changes in the organization of the cells within the tubules first became visible by 10 dpp in the *Dnmt3L*^-/- ^males, although there was no definite defect (data not shown). By 12 dpp, clear differences between control (*Dnmt3L*^+/+ ^and *Dnmt3L*^+/-^) and *Dnmt3L*^-/- ^testes could be noted: fewer cells appeared to enter meiosis and almost no cells with condensed nuclei were visible. As exemplified in Fig. 1A, very few tubules display cells engaged in meiosis in the absence of DNMT3L at 12 dpp.

### Reduced germ cell numbers in DNMT3L-deficient newborn males

These histological observations prompted us to quantify the number of germ cells present in control and DNMT3L-deficient testes using the germ cell-specific antibody GCNA1. GCNA1 can be detected as early as embryonic day (E) 11.5 and is expressed in all germ cells until the zygotene stage, after which point expression becomes restricted to specific chromatin domains in pachytene spermatocytes and round spermatids in the postnatal testis [39]. First, we established that there were no differences in germ cell counts between *Dnmt3L*^+/+ ^and *Dnmt3L*^+/-^males at 10 dpp (data not shown). Subsequently, *Dnmt3L*^+/- ^males were used as control. Staining of *Dnmt3L*^+/- ^testes at 10 dpp revealed numerous cells, but there were consistently fewer cells stained in *Dnmt3L*^-/- ^testes (Fig. 1B – top panels); the same was observed at 8 dpp (Fig. 1B – bottom panels). In fact, there was a clear difference in germ cell counts as early as day 6 (Fig. 1C). A trend to decreased counts could also be noted at 4 dpp, but there were only two males per group available for analysis. Clearly, absence of *Dnmt3L *causes an early mitotic defect that results in a lower pool of germ cells able to engage in spermatogenesis.

### Delayed entry of germ cells into meiosis

**Figure 2:**
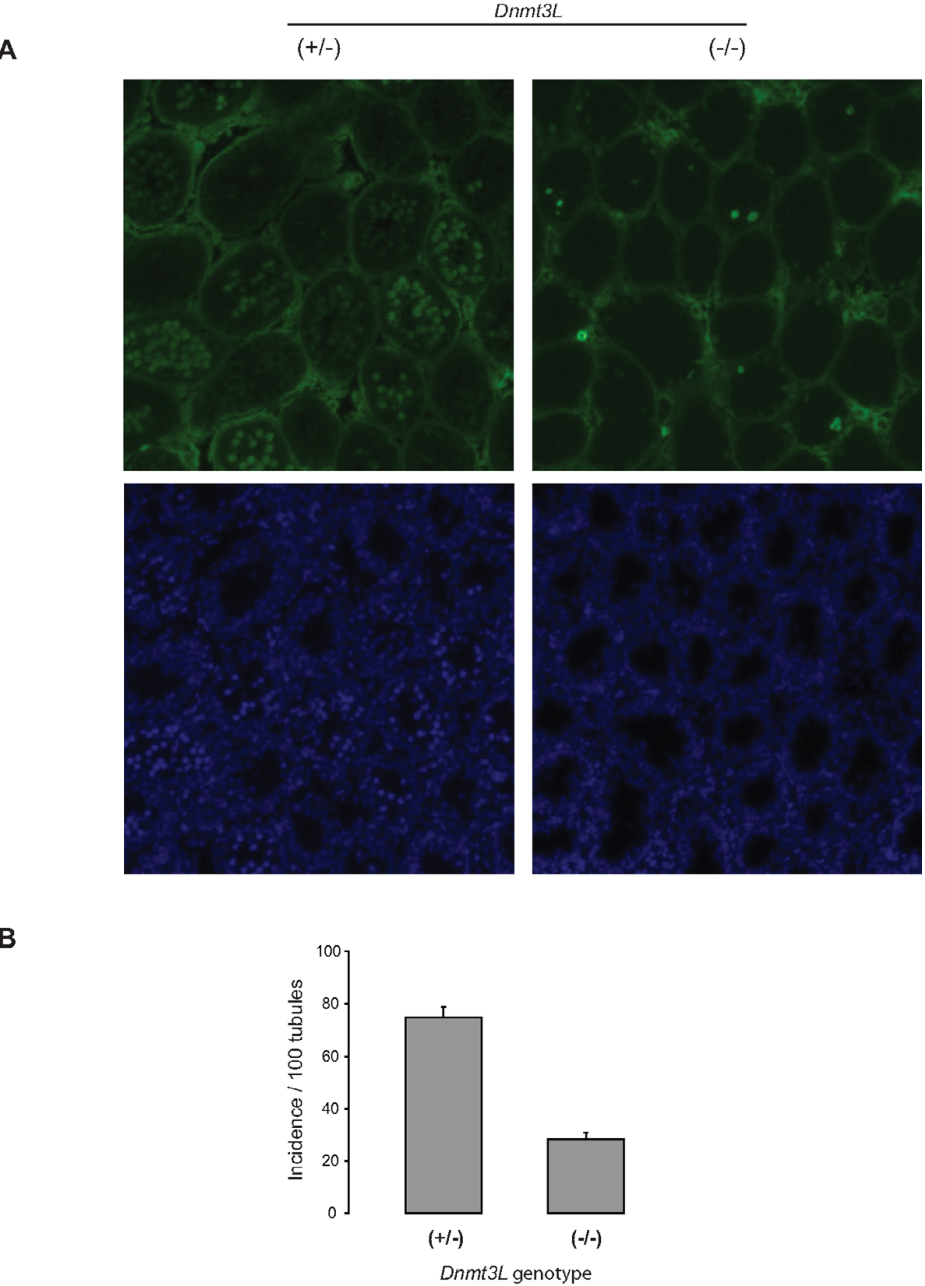
**Delayed onset of meiosis in the absence of DNMT3L**. Immunofluorescence analysis of testis cross-section from 10 dpp *Dnmt3L*^+/- ^and *Dnmt3L*^-/- ^mice using an antibody directed against γ-H2AX (green). **A) **(Top panel – left) Representative staining pattern obtained in *Dnmt3L*^+/- ^sections: a majority of tubules contain γ-H2AX positive cells. (Top panel- right) Only a few tubules containing positive cells can be detected in *Dnmt3L*^-/- ^sections. Below each panel are the corresponding DAPI (blue) counterstained images. **B) **Quantification of the number of tubules containing at least one positive γ-H2AX cell in *Dnmt3L*^+/- ^and *Dnmt3L*^-/- ^testes. The incidence of positive tubules per 100 tubules was determined from six to nine sections per male, with three males per genotype group. Results are presented as means ± SEM.

Our histological analysis revealed that fewer cells were in meiosis at 12 days postpartum (Fig. 1A). To analyze progression to meiosis, we examined the presence of phosphorylated histone H2AX (known as γ-H2AX) positive cells in *Dnmt3L*^-/- ^males; γ-H2AX is recruited to sites of double-stranded breaks and thus serves as an early marker of entry into meiosis [40]. Since the first meiotic cells become visible between 9–10 dpp [38], we counted the number of tubules that contained at least one positive γ-H2AX cell in 10 dpp *Dnmt3L*^+/- ^and *Dnmt3L*^-/- ^testes. As shown in Figure 2A, numerous tubules containing at least one positive cell (usually ≥ 5 cells/tubule) were observable in *Dnmt3L*^+/- ^males, while only a few tubules presented positive cells (usually ≤ 5 cells/tubule) in *Dnmt3L*^-/- ^males. The incidence of positive tubules, i.e. tubules presenting at least one γ-H2AX positive cell, was calculated per 100 tubules for both genotypes (Fig. 2B). There were 74.9 ± 4.0 positive tubules in *Dnmt3L*^+/- ^males compared to only 28.2 ± 2.6 in *Dnmt3L*^-/- ^males. Thus, in addition to depletion in number of germ cells, these results also suggest that there is a delay in the onset of meiosis in *Dnmt3L*-deficient males.

### Developmentally regulated expression of Dnmt3L during spermatogenesis

These findings, coupled to the fact that a number of groups have reported exclusive expression of *Dnmt3L *in pre- and peri- natal gonocytes [21,22,24,31] while others have also detected *Dnmt3L *in more mature cell types [23], prompted us to clarify the timing of *Dnmt3L *expression during spermatogenesis. We used quantitative RT-PCR (qRT-PCR) to determine the expression of *Dnmt3L *in postnatal male germ cells obtained by sedimentation at unit gravity and flow cytometry; in all, nine different populations enriched in specific germ cell types could be isolated using mice of different ages. As described by Dym and colleagues [41], type A spermatogonia isolated from 6-day-old mouse testes are a mixture of 3 subtypes of type A spermatogonia, namely dark (subtype I), transitional (subtype II) and pale (subtype III) spermatogonia. In contrast, 8-day-old mouse testes contain more mature type A spermatogonia as well as type B spermatogonia [38]. For reasons of simplicity and to allow for distinction with the type A spermatogonia isolated from 8-day-old testes, day-6 spermatogonia will be referred to as primitive type A spermatogonia.

**Figure 3:**
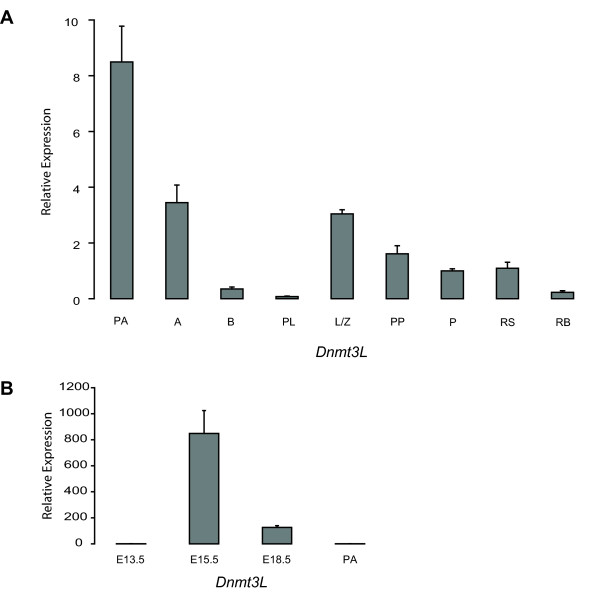
**Dynamic expression of *Dnmt3L *in male germ cells**. **A) **Relative quantification of *Dnmt3L *expression in postnatal male germ cells. Real-time RT-PCR was used to determine the expression profile of *Dnmt3L *in primitive type A (PA), type A (A) and type B (B) spermatogonia, preleptotene (PL), leptotene/zygotene (L/Z), prepubertal pachytene (PP) and pachytene (P) spermatocytes, as well as round spermatids (RS) and residual bodies/elongating spermatids (RB). Expression was determined in triplicate in each of the two series of germ cells and normalized to *18S *expression; normalized results were calibrated to expression in pachytene spermatocytes. Shown here are the mean expression results obtained for one series. Mean ± SD. **B) **Relative quantification of *Dnmt3L *expression in prenatal male germ cells. Quantitative RT-PCR was used to measure the expression levels of *Dnmt3L *in total RNA extracted from E13.5, E15.5 and E18.5 prospermatogonia and 6 dpp primitive type A spermatogonia (PA). Expression of *Dnmt3L *was determined in triplicate in each of the two series of germ cells and normalized to *18S *expression; normalized values were calibrated to the expression found in E13.5 gonocytes. Shown here are the mean expression results obtained for one series. Mean ± SD.

*Dnmt3L *could be detected in all cell types tested, albeit at different levels (Fig. 3A). Expression was highest in primitive type A spermatogonia and remained elevated in type A spermatogonia, but decreased drastically in type B spermatogonia and was at its lowest in preleptotene spermatocytes. *Dnmt3L *was found in leptotene/zygotene spermatocytes at levels similar to those seen in type A spermatogonia, but expression decreased steadily as meiosis progressed. *Dnmt3L *expression was also detected in round spermatids and residual bodies/elongating spermatids, but at low levels.

Next, we assessed *Dnmt3L *expression before, during and after the initial period of DNA methylation acquisition in the fetal male germ line. We isolated pure populations of gonocytes by flow cytometry at E13.5, E15.5 and E18.5, and used qRT-PCR to analyze the expression of *Dnmt3L *in these cells. Expression peaked in E15.5 gonocytes, being ~800 times higher than in E13.5 gonocytes (Fig. 3B). *Dnmt3L *was still present at high levels in E18.5 gonocytes, but by this developmental stage, expression was ~8-fold lower than that seen at E15.5. For comparison purposes, the expression result obtained for postnatal primitive type A spermatogonia was reanalyzed in the context of the prenatal expression results, showing that *Dnmt3L *expression in these cells is ~800 times lower than in E15.5 gonocytes. And since expression of *Dnmt3L *in pachytene spermatocytes is approximately eight times lower than in primitive type A spermatogonia, it is therefore close to 6400 times lower than in E15.5 gonocytes. The expression profiles presented here demonstrate that, although *Dnmt3L *expression is at its highest before birth, it is still detectable throughout postnatal male germ cell development but at considerably lower levels.

### DNA methylation analysis of Dnmt3L mutant testes

Thus far, DNMT3L has been implicated in the methylation of paternally imprinted genes and interspersed retrotransposons in male germ cells [20,22,24]. Another study has also revealed a number of changes in gene expression in whole testes lacking DNMT3L [36]. While a delay in spermatogenesis progression may be responsible for these expression differences, they may also suggest that this protein is involved in methylation of other sequences. Although few techniques allow for the analysis of methylation differences at the whole genome level in a quantitative manner, restriction landmark genomic scanning (RLGS) allows us to scan and quantify the methylation status of approximately 2600 genomic NotI restriction sites. These sites are found mostly within CpG islands but are also located in non-coding unique and repetitive sequences outside of CpG islands. Presence or absence of a spot on an RLGS profile is indicative of the methylation state of that site. If the restriction site is unmethylated, NotI will cleave the DNA template, resulting in appearance of a spot on the scan; conversely, no spot will be visible if the site is methylated.

**Figure 4:**
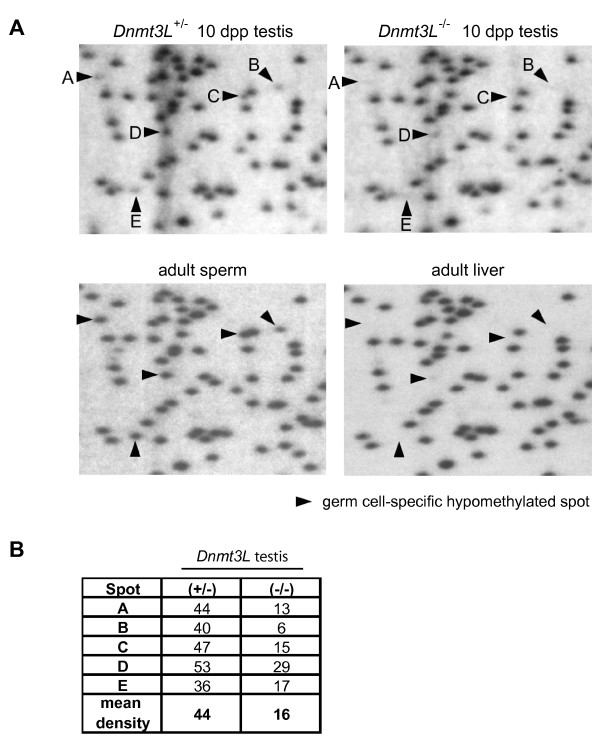
**RLGS analysis of *Dnmt3L*-deficient testis**. Restriction landmark genomic scanning was used to investigate global methylation levels of *Dnmt3L *mutant testis. The following tissues were used for analysis: 10 dpp whole testes from *Dnmt3L*^+/- ^and *Dnmt3L*^-/- ^males, and sperm and liver from *Dnmt3L*^+/+ ^adult males. **A) **Shown here are enlargements of autoradiographs obtained for these tissues. Arrowheads point to five pre-identified germ cell-specific spots present in adult sperm but absent in liver [42]. **B) **Densitometry analysis of the five germ cell-specific spots in *Dnmt3L*^+/- ^and *Dnmt3L*^-/- ^testes. Once the somatic-to-germ cell ratio of a 10 dpp testis is considered and the lower germ cell counts in *Dnmt3L*^-/- ^testes are accounted for, the mean density values obtained for *Dnmt3L*^+/- ^and *Dnmt3L*^-/- ^10 dpp testes are reflective of the proportion of germ cells present in these tissues, not of changes in methylation state. Potential changes in DNA methylation levels in *Dnmt3L*^-/- ^testes fall under the limit of detection.

In an attempt to find additional targets of DNMT3L, RLGS was performed on whole testes collected from 10 dpp *Dnmt3L *males of all three genotypes. We first compared the methylation profiles of *Dnmt3L*^+/+ ^and *Dnmt3L*^+/- ^testes and found them to be identical (data not shown). Next, we compared the *Dnmt3L*^+/- ^profile to the one obtained for *Dnmt3L*^-/- ^testis and found striking differences between genotypes, as shown in Figure 4A (top panels). The intensity of a number of spots appeared to decrease in the absence of DNMT3L, suggesting that these sites were becoming methylated. To identify the spots that were changing between *Dnmt3L*^+/- ^and *Dnmt3L*^-/- ^testes, we compared these profiles to the ones obtained for adult sperm and liver (Fig. 4A-bottom panels). In a previous study, we identified a number of differentially methylated sites between mature sperm and adult somatic tissues, including liver [42]. All the spots that were changing in *Dnmt3L*^-/- ^testis were the germ cell-specific ones identified in mature sperm. This observation prompted us to question the legitimacy of the methylation differences observed in whole testis of *Dnmt3L*^-/- ^males. A testis at day 10 after birth is made up of approximately 40% germ cells, the rest being somatic cells [38]. Measuring the density of the five changing spots labeled in Figure 4A gave an average density value of 44 and 16 for *Dnmt3L*^+/- ^and *Dnmt3L*^-/- ^testes, respectively (Fig. 4B). After considering the proportion of germ cells present in a testis at 10 dpp and the decrease in germ cell counts observed at this point in *Dnmt3L*^-/- ^testes (Fig. 1C), it became obvious that the mean density values corresponded roughly to the amount of germ cells present in these tissues. Therefore, changes in spot density observed in the *Dnmt3L*^-/- ^testis were due to the loss of germ cells in the absence of DNMT3L, not to changes in DNA methylation state. These results render the use of whole testes for gene expression or DNA methylation analyses invalid and further emphasize the need to use isolated populations of male germ cells.

### Requirement of DNMT3L for the correct establishment of wide-spread DNA methylation patterns

To isolate *Dnmt3L*-deficient male germ cells, we crossed *Dnmt3L*^+/- ^mice with mice expressing green fluorescent protein (GFP) in the germ line [43]. By crossing [GFP^+^, *Dnmt3L*^+/-^] males and females together, primitive type A spermatogonia could be isolated by flow cytometry at 6 dpp from *Dnmt3L *(+/+), (+/-) and (-/-) GFP^+ ^males. We chose to isolate cells at this time to obtain a maximal number of germ cells, as spermatogonia are actively dividing at this stage and GFP is still expressed highly in these cells. DNA methylation analyses were then conducted on these pure populations of primitive type A spermatogonia. Quantitative analysis of DNA methylation using real-time PCR, or qAMP, was used to evaluate the methylation state of a number of sequences, including imprinted genes and whole chromosomes. The qAMP assay measures the percentage of CpG dinucleotide methylation within a target sequence by means of real-time PCR amplification of DNA templates digested with methylation-sensitive and methylation-dependent restriction enzymes [44].

**Figure 5:**
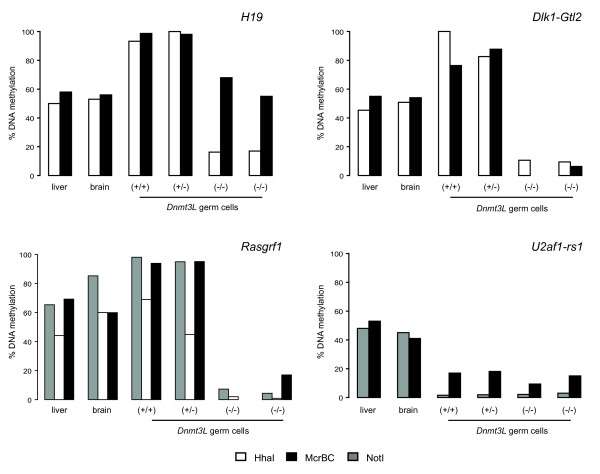
**Abnormal methylation of paternally imprinted genes in *Dnmt3L*^-/- ^germ cells**. DNA extracted from the purified populations of *Dnmt3L *germ cells was analyzed via a quantitative restriction enzyme assay, quantitative analysis of methylation PCR or qAMP [44]. Analysis of established DMR regions of three known paternally methylated imprinted genes, *H19 *(top left), *Dlk1-Gtl2 *(top right) and *Rasgrf1 *(bottom left), and one known maternally methylated imprinted gene, *U2af1-rs1 *(bottom right). At least one site of *H19, Dlk1-Gtl2 *and *Rasgrf1 *is affected in the two *Dnmt3L*^-/- ^samples analyzed, whereas *U2af1-rs1 *methylation remains unchanged. Three different enzymes were used in qAMP: HhaI (white), McrBC (black) and NotI (grey).

First we used qAMP to confirm that paternally imprinted genes were not acquiring methylation marks properly in *Dnmt3L*^-/- ^cells. Three paternally imprinted genes (*H19*, *Dlk1-Gtl2 *and *Rasgrf1*) and one maternally imprinted gene (*U2af1-rs1*) were chosen for analysis. Primers were designed to the established differentially methylated region (DMR) of these genes [45-48]. Two somatic tissues serving as positive controls were analyzed in parallel with *Dnmt3L *mutant germ cells. In both liver and brain, we obtained an average of 50% methylation for *H19*, *Dlk1-Gtl2 *and *U2af1-rs1*, as expected for somatic tissues (Fig. 5). In *Dnmt3L*^+/+ ^and *Dnmt3L*^+/- ^germ cells, methylation levels of *H19*, *Dlk1-Gtl2 *and *Rasgrf1 *neared 100% as expected for paternally imprinted genes, while methylation of the maternally imprinted gene *U2af1-rs1 *was almost undetectable (Fig. 5). In *Dnmt3L*^-/- ^germ cells, *U2af1-rs1 *methylation levels remained low, showing that hypomethylated sequences do not gain abnormal methylation marks in absence of DNMT3L. However, we did observe methylation differences for *H19*, *Dlk1-Gtl2 *and *Rasgrf1 *in these same deficient germ cells. For *H19 *(Fig. 5 – top left), the HhaI site had lost ~85 % of its methylation, while the McrBC sites were demethylated by about 40%; for *Dlk1-Gtl2 *and *Rasgrf1 *(Fig. 5 – top right and bottom left), methylation was down to less than 10% at all cut sites tested. Our results for *H19 *are in agreement with the mosaic pattern obtained by bisulphite sequencing reported by others [22,24], while hypomethylation of the *Dlk1-Gtl2 *and *Rasgrf1 *loci differs from previous studies that were performed at later stages of testis development [20,22]. Thus, we find that paternally imprinted genes do not acquire proper methylation marks in cells lacking DNMT3L.

**Figure 6:**
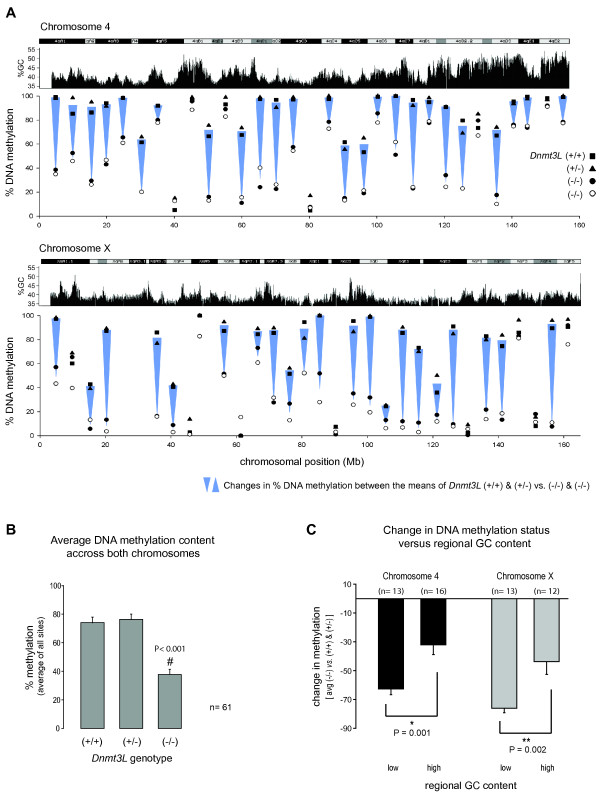
**Decreased levels of DNA methylation on chromosomes 4 and X in *Dnmt3L*^-/- ^germ cells**. **A) **DNA methylation analysis of non-CpG island DNA on chromosomes 4 (top) and X (bottom) using qAMP. The percent of DNA methylation of each amplified region is shown for *Dnmt3L*^+/+ ^(solid square), *Dnmt3L*^+/- ^(solid triangle) or *Dnmt3L*^-/- ^(open and solid circles) male germ cells; differences in methylation are depicted by blue arrows. DNA methylation differences are illustrated in the context of chromosomes 4 and X ideograms and regional GC content (adapted from the UCSC genome browser). For both chromosomes, most sites examined showed lower levels of DNA methylation in the absence of DNMT3L. **B) **Average DNA methylation levels across all sites tested on chromosomes 4 and X. For the 61 sites tested on both chromosomes, the overall DNA methylation level in *Dnmt3L*^-/- ^germ cells decreased by more than 37% (P < 0.001) as compared to *Dnmt3L*^+/+ ^and *Dnmt3L*^+/- ^germ cells. **C) **Influence of the regional GC content on the changes in DNA methylation status observed in *Dnmt3L*^-/- ^germ cells. The methylation level of a given locus was compared to the regional GC content of 50kb of flanking sequence; the GC content of a region was characterized as being "low" or "high" if it was, respectively, below or above the average GC content of the analyzed chromosome. The number of sites included in the analysis is indicated in the relevant GC content category for each chromosome. For both chromosomes, loci present in lower GC content regions were significantly more demethylated than loci found in areas of higher GC density (Chr4, P = 0.001; ChrX, P = 0.002). Data are presented as mean +/- SEM.

Staining DNA gels with ethidium bromide showed that the genome of *Dnmt3L*-deficient germ cells is demethylated [22]. We extended our analysis of DNA methylation levels to multiple sites along an autosome and a sex chromosome in *Dnmt3L *mutant germ cells to determine if other sequences were demethylated and contributing to genomic hypomethylation. Chromosomes 4 and X were chosen for analysis using qAMP. Primers were designed to flank HhaI, HpaII or McrBC restriction sites and were placed at roughly five Mb intervals along each chromosome. Loci were chosen solely on the basis of the sequence not being in proximity of a known 5' region of a gene, a CpG island or any type of repetitive sequence; primers were specifically designed to intronic-intergenic sites as the effect of DNMT3L deficiency on interspersed repeat sequences had already been shown by others [22,24]. Approximately 30 small regions were surveyed along each chromosome. As illustrated in Figure 6A and 6B, striking differences in DNA methylation levels could be observed for both chromosomes between *Dnmt3L*^+/+ ^and *Dnmt3L*^+/- ^cells and the two *Dnmt3L*^-/- ^samples. Most sites presented decreased DNA methylation levels, albeit to different extents. As it was the case for the maternally imprinted gene *U2af1-rs1 *(Fig. 5), hypomethylated loci remained unmethylated in the absence of DNMT3L. When methylation levels across the 61 sites tested along both chromosomes were averaged, a significantly (P < 0.001) lower level of methylation was detected in *Dnmt3L*^-/- ^germ cells (37.7% ± 3.7%) as compared to *Dnmt3L*^+/+ ^(74.0% ± 4.0%) and *Dnmt3L*^+/- ^(76.3% ± 3.7%) (Fig. 6B).

### Relationship between DNMT3L-targeted DNA methylation and the regional GC content

Interestingly, on each chromosome some loci appeared to lose very little methylation and the methylation status of some hypermethylated loci did not change at all. We observed that those loci that were acquiring their methylation properly in the absence of DNMT3L were situated in regions of high GC content, while those loci that failed to gain their methylation properly in *Dnmt3L*^-/- ^germ cells were located in lower GC content regions (Fig. 6A). To test the possible relationship between methylation acquisition and GC content in the presence or absence of DNMT3L, we compared the DNA methylation levels of all tested chromosomal loci to the regional GC content of 50 kb of flanking DNA sequence (Fig. 6C). Loci were categorized as being in a "low" or "high" GC content area according to their %GC being below or above the average GC content of the analyzed chromosome; the average GC content of chromosomes 4 and X are 42.3% and 39.2%, respectively [49]. Because the amount of DNA methylation in *Dnmt3L*^-/- ^germ cells at a given locus was being compared to the amount of DNA methylation observed in *Dnmt3L*^+/+ ^and *Dnmt3L*^+/-^, we removed the normally unmethylated sites (< 15% DNA methylation) in *Dnmt3L*^+/+ ^and *Dnmt3L*^+/- ^from the analysis, as very little to no loss could occur at these loci (two sites for Chr4; five for ChrX). And, since sites that are partially methylated in *Dnmt3L*^+/+ ^and *Dnmt3L*^+/- ^germ cells have less methylation to lose, the amount of DNA methylation lost in *Dnmt3L*^-/- ^germ cells was considered as a proportion of the amount of DNA methylation detected in *Dnmt3L*^+/+ ^and *Dnmt3L*^+/- ^germ cells for each locus tested. Interestingly, loci found in lower GC-content areas were significantly more hypomethylated than sites found in GC-rich regions in *Dnmt3L*^-/- ^germ cells (Chr4, P = 0.001; ChrX, P = 0.002). For chromosome 4, sites found in GC-rich areas only lost 32.1% ± 6.8% of their methylation versus 62.7% ± 3.9% for sites found in GC-poor areas. The situation was similar for chromosome X, where sites in lower GC-content region lost 76.0% ± 3.1% of their methylation against only 43.8% ± 8.9% for sites in GC-rich areas. The data presented here suggest that the role of DNMT3L extends to a larger part of the genome than was previously reported and is influenced by the regional GC content of a locus in addition to the nature of the sequence.

### Lack of Dnmt up- or down- regulation in Dnmt3L-deficient germ cells

**Figure 7:**
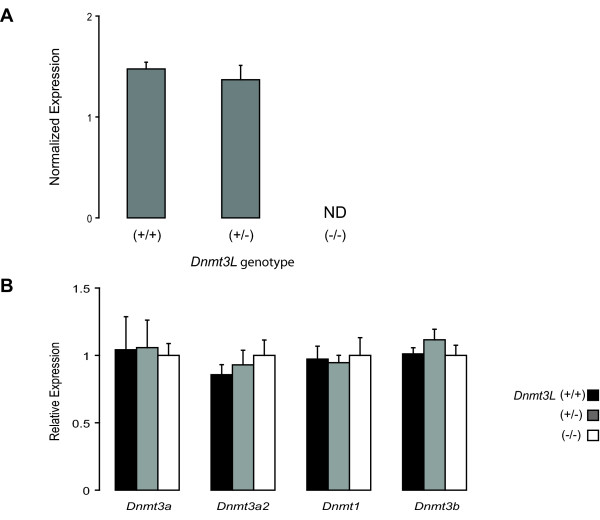
**Expression of DNA methyltransferases in *Dnmt3L*^-/- ^male germ cells**. QRT-PCR was used to evaluate the relative expression of *Dnmt *genes in isolated *Dnmt3L *mutant 6 dpp primitive type A spermatogonia. Expression of a given *Dnmt *was analyzed in triplicate and normalized to *18S *expression for each genotype; the normalized value was calibrated to the expression observed in *Dnmt3L*^-/- ^cells. **A) **Illustration of *Dnmt3L *expression in *Dnmt3L*^+/+^, *Dnmt3L*^+/- ^or *Dnmt3L*^-/- ^male germ cells. *Dnmt3L *could not be detected in the *Dnmt3L*^-/- ^sample. Only the normalized values are presented in this case, calibrating the data to the *Dnmt3L*^-/- ^sample being impossible. ND, not detectable. **B) **Expression of *Dnmt3a*, *Dnmt3a2*, *Dnmt3b *and *Dnmt1 *in *Dnmt3L*^+/+ ^(black bars), *Dnmt3L*^+/- ^(grey bars) and *Dnmt3L*^-/- ^(white bars) germ cells. No observable differences in DNA methyltransferase expression could be detected in *Dnmt3L*-deficient germ cells at 6 dpp. Results from one series of germ cells are presented as mean ± SD.

Finally, we investigated whether the expression of other *Dnmts *changed in response to DNMT3L depletion. In an earlier study conducted on 15 dpp *Dnmt3L*^-/- ^oocytes, we observed an up-regulation in expression of *Dnmt3b *and *Dnmt1o *(the oocyte-specific form of *Dnmt1*) transcripts in absence of DNMT3L [50]. Up-regulation in expression of a given *Dnmt *gene could partly explain how some sequences retain their methylation. Real-Time RT-PCR was used to examine the levels of *Dnmt3a*, *Dnmt3a2*, *Dnmt3b *and *Dnmt1 *in *Dnmt3L*^+/+^, *Dnmt3L*^+/- ^and *Dnmt3L*^-/- ^germ cells isolated as previously described. We first evaluated *Dnmt3L *expression in our three germ cell samples (Fig. 7A). As anticipated, *Dnmt3L *transcripts could not be detected in *Dnmt3L*^-/- ^cells. Interestingly, expression in *Dnmt3L*^+/- ^cells was similar to that in *Dnmt3L*^+/+ ^cells. Next, we determined the levels of the other *Dnmts *in these same cells (Fig. 7B). There were no observable differences in expression of any of the *Dnmts *or their transcript variants in absence of *Dnmt3L*.

## Discussion and conclusion

Proper establishment and propagation of DNA methylation patterns during male germ cell development are crucial to transmission of epigenetic information to the next generation. The nature of the mechanisms governing the creation of these patterns has started to emerge as recent studies have pointed to DNMTs that could be involved in this process [20,21,23]. A number of gene targeting experiments have revealed the importance of *Dnmt3L *to spermatogenesis: some paternally imprinted genes and retrotransposons are abnormally methylated in deficient germ cells and these cells are unable to progress through meiosis [20,22,24]. However, how and why a protein incapable of directly methylating DNA causes such a drastic effect on germ cell integrity is still unclear. We recently conducted a preliminary study on isolated *Dnmt3L *mutant male germ cells and showed that loci identified to be specifically methylated in sperm, and not in somatic tissues, were not methylated properly in absence of DNMT3L [42]. Here, we expand on these findings and provide evidence that DNMT3L is involved in broader DNA methylation events possibly by targeting methylation to lower GC content regions. We also reveal the presence of a mitotic defect in *Dnmt3L *mutant males, as germ cell counts are already significantly lower at 6 days after birth, in addition to showing that the rate of entry into meiosis is markedly reduced in *Dnmt3L*^-/- ^germ cells. Finally, we present a comprehensive developmental expression profile of *Dnmt3L *in isolated male germ cells and show that this gene is still expressed during postnatal spermatogenesis.

### Mitotic defect and delayed entry into meiosis in absence of DNMT3L

Detailed histological analysis of early testicular development in *Dnmt3L *mutant males has allowed a better definition of the timing of the histopathological defects observed in mice lacking DNMT3L. Although testis histology appears normal, *Dnmt3L*^-/- ^males do present abnormalities at one week of age: germ cell counts are down by more than 50% at 6 dpp. Hata and colleagues [36] recently demonstrated that at 3 weeks of age germ cells from *Dnmt3L*^-/- ^males proliferate at about half the rate of *Dnmt3L*^+/+ ^germ cells. Proliferation rates could be affected from the beginning, explaining why germ cell counts are already affected at 4 dpp – the time at which most germ cells resume mitosis.

In addition to lower germ cell counts, we also observed a delay in entry into meiosis, as illustrated by the lower frequency of tubules containing γ-H2AX positive cells in 10-day-old DNMT3L-deficient male mice. Commitment of spermatogonia to meiosis is an important step in germinal differentiation upon which point dedifferentiation becomes impossible. Germ cells have to express the proper set of genes and present the appropriate chromatin structure that will allow for their passage through meiosis [33]. Delay in onset of meiosis in *Dnmt3L *mutant males could be caused by failure of germ cells to undergo the necessary steps required to proceed to the next developmental stage. As DNA methylation and histone modifications are interconnected, a genome-wide DNA methylation defect could cause chromatin structure abnormalities, retarding entry into meiosis. In fact, atypical chromatin structures have been reported in *Dnmt3L*^-/- ^germ cells from the intermediate spermatogonia stage all the way through zygonema [24]. Transcriptional reactivation of retroviral elements and their subsequent random reintegration could also contribute to delaying entry in meiosis by compromising the integrity of the genome of *Dnmt3L*^-/- ^germ cells through *de novo *mutations or ectopic gene expression [22,24].

### Expression dynamics of Dnmt3L during male germ cell development

Acquisition of methylation patterns during male germ cell development is multifaceted: the process begins prenatally in gonocytes and continues after birth up until the end of prophase I of meiosis [51]. Additionally, acquired marks are maintained as DNA replication takes place in spermatogonia and preleptotene spermatocytes. We recently demonstrated that expression of *Dnmt3a *and *Dnmt3b*, the postulated *de novo *DNA methyltransferases, is tightly regulated during spermatogenesis [19]. Here, we investigated expression of the full-length form [52] of the DNA methyltransferase 3-Like gene, *Dnmt3L*, during both prenatal and postnatal male germ cell development. Our analyses conducted on pure populations of prenatal gonocytes revealed strikingly elevated levels of this transcript during the initial period of methylation acquisition, as previously reported [18,21-24]. In agreement with previous findings by Hata *et al*. [23], we also found expression of *Dnmt3L *during spermatogenesis. However, the magnitude in expression of *Dnmt3L *in postnatal male germ cells was not comparable to the one observed before birth, being approximately 6400 times lower in pachytene spermatocytes than in E15.5 gonocytes, explaining how it could have gone undetected by others. Probing the postnatal expression of *Dnmt3L *revealed a profile very reminiscent of the one obtained for *Dnmt1*, *Dnmt3a *and *Dnmt3b *in isolated populations of male germ cells [19]. The data presented in Figure 3A show that *Dnmt3L *expression is down-regulated during the same two developmental windows identified for other *Dnmts*, i.e. differentiation of spermatogonia into spermatocytes and pachynema. These results are the first indication of regulated expression of *Dnmt3L *during postnatal male germ cell development. DNMT3L could be involved in the establishment or the maintenance of methylation patterns during postnatal male germ cell development, in addition to the role it plays in gonocytes.

### Genome-wide demethylation observed in germ cells lacking Dnmt3L and link with the regional chromatin state

Performing qAMP on primitive type A spermatogonia isolated from *Dnmt3L *mutant males allowed us to test the methylation status of a number of sequences. When the status of paternally imprinted genes was assessed, we observed loss of methylation at the *H19 *DMR similarly to what has already been published, but for the other imprinted genes, we generally found a higher reduction in DNA methylation levels compared to former studies [20,22,24]. The use of different enzymes to perform the methylation-sensitive enzymatic digestions for Southern Blot analysis could potential provide a different answer as different CpGs are being analyzed. However, slight differences in the localization of the primers or probes used to examine the various regions would most likely explain these inconsistencies. As well, we believe that the discrepancies can also be partly accounted for by differences in timing of sample collection and techniques employed to isolate the cells. The analyses presented here were carried out on pure preparations of primitive type A spermatogonia isolated at 6 dpp, the earliest time point tested for DNA methylation differences in a *Dnmt3L *mutant mouse model, while all other studies have been conducted on mixed populations of germ cells isolated at later time points [20,22,24]. This discrepancy between early and late stages of spermatogenesis may indicate the existence of compensatory mechanisms acting during spermatogonial proliferation to partially restore methylation patterns at paternally imprinted genes in a DNMT3L-independent manner.

The methylation state of multiple loci on both an autosome and a sex chromosome was also analyzed using qAMP. Loci analyzed by qAMP were selected solely on the basis of not being in the vicinity of a gene or a repeat element and were spaced at roughly 5Mb intervals, allowing for an unbiased screen. Numerous sites across chromosomes 4 and X were severely demethylated in *Dnmt3L*^-/- ^germ cells. Again, hypomethylated sequences remained so in cells lacking DNMT3L, suggesting that inherited methylation patterns are erased properly in primordial germ cells prior to DNA methylation reprogramming and that methyl groups are not redistributed to ectopic genomic positions. Interestingly, some loci had very little methylation, while others had the same levels of methylation as found in *Dnmt3L*^+/+ ^and *Dnmt3L*^+/- ^germ cells. We initially hypothesized that either these sequences were not targets of DNMT3L and were methylated without requiring DNMT3L stimulation, or *Dnmt3L *inactivation caused for an up-regulation in the expression of other *Dnmt *genes to compensate for the loss of DNMT3L. When we monitored expression of other *Dnmt *genes in *Dnmt3L*^-/- ^male germ cells we did not detect any difference in *Dnmt *expression, suggesting that *Dnmt *up-regulation is not a response mechanism associated with the inactivation of *Dnmt3L *in the male germ line. This differs with what we previously observed in *Dnmt3L*^-/- ^oocytes [50], suggesting that the regulatory mechanisms governing expression of DNA methyltransferases in the male and female germ lines differ. Evidence of this is seen for *Dnmt1*, as different mechanisms appear to control DNMT1 down-regulation during male and female meiosis [18]. However, a detailed developmental study is still required before this possibility can be excluded, as compensatory mechanisms could be activated at other times during male germ cell development.

When broad regional characteristics of chromosomes were taken into account, namely the GC content, we found a statistically significant relationship between regions of lower GC density and DNA methylation aided by DNMT3L. Whereas DNMT3L is probably not responding to the regional GC content per se, our findings indicate that broader regional chromatin characteristics may be dictating where DNMT3L directs DNA methylation by recognizing specific histone modifications or interacting with specific histone modification enzymes and their associated partners. Functionally, GC-poor regions are usually found in heterochromatic Giemsa bands and are characterized by a lower frequency of genes, a lower rate of transcription and later replication timing [49]. Heterochromatin is generally decorated by specific histone modifications such as methylation of lysine residues K9 and K20 on histones H3 and H4, respectively [53]. While a functional relationship between DNA and histone H3K9 methylation was first established in *Neurospora crassa *[54], recent data showed that DNMT3L binds specifically to the N-terminal tail of H3 when lysine 4 was unmethylated and is insensitive to the methylation state of lysine 9 [55]. These data indicate that DNMT3L could recruit or activate DNMT3a2 and DNMT3b upon contact with nucleosomes that contain unmethylated H3K4. Conversely, the wide-spread lack of methylation observed here could be secondary to the lack of methylation at retrotransposable elements: methylation of intergenic-intronic loci could be acquired as a consequence of spreading of methylation to neighbouring sequences from retrotransposons. Hypomethylation of these loci could still be contributing to the phenotype by altering gene expression patterns or modifying global chromatin architecture. Additional studies performed on isolated prenatal gonocytes should allow these various possibilities to be addressed.

## Methods

### Mice

CD-1 mice were purchased from Charles River Canada Inc. (St-Constant, QC, Canada). *Dnmt3L *mutant mice [21] and GOF18/deltaPE-Oct-4/GFP transgenic mice [43] have been described elsewhere. Noon of the day on which the vaginal plug was found was designated as embryonic day (E) 0.5, while day of birth was designated as postpartum day (dpp) 0. All procedures were performed in accordance with the Canadian Council on Animal Care and approved by the McGill University Animal Care Committee.

### Isolation of Male Germ Cells by Flow Cytometry

Timed-pregnancies were established between CD-1 females and GOF18/deltaPE-Oct-4/GFP males. Testes were collected from male embryos at E13.5, E15.5 and E18.5 or from male pups at 6 dpp and rinsed twice in sterile phosphate buffered saline (PBS). Decapsulated testes were digested in 0.25% trypsin-EDTA (Gibco-BRL/Invitrogen, Burlington, ON, Canada) for 10 minutes at 37°C, dispersed and digested further for 10 minutes. The cell suspension obtained was washed twice and resuspended in PBS. GFP-positive gonocytes and primitive type A spermatogonia were collected by flow cytometry using a MoFlow cell sorter (Cytomation Inc., Ft. Collins, CO).

### Isolation of Male Germ Cells by Sedimentation Velocity

Purified populations of male germ cells were obtained from the testes of 8-, 17- and 70-dpp CD-1 mice according to the sedimentation velocity cell separation method as described previously [19,56]. Cells were identified on the basis of morphological criteria and size. Populations of type A spermatogonia (average purity = 86%) and type B spermatogonia (average purity = 83%) were obtained from the testes of 8dpp mice (n = 2 cell separations); preleptotene spermatocytes (average purity = 85%), leptotene/zygotene spermatocytes (average purity = 87%) and prepubertal pachytene spermatocytes (average purity = 80%) were obtained from the testes of 17dpp mice (n = 2 cell separations); finally, pachytene spermatocytes (average purity = 81%), round spermatids (average purity = 88%) and elongating spermatids mixed with residual bodies (average purity = 86%) were obtained from 70dpp mice (n = 2 cell separations).

### Quantitative RT-PCR

Total RNA was extracted from snap-frozen pellets of male germ cells using the RNeasy Mini kit with DNaseI treatment according to the manufacturer's protocol (Qiagen Inc., Mississauga, ON, Canada). Real-Time or quantitative RT-PCR (qRT-PCR) was performed using the Mx4000 qPCR system from Stratagene (La Jolla, CA) using the QuantiTect ™ SYBR ^® ^Green RT-PCR kit (Qiagen) as described previously [18]. The primers used to determine the relative expression of *Dnmt1*, *Dnmt3a*, *Dnmt3a2*, *Dnmt3b *and *Dnmt3L *according to the standard curve method have been described elsewhere [18,19,57]. Note that the primers assaying *Dnmt3L *expression were designed to pick up the prospermatogonia (full-length) form of *Dnmt3L *and not any of the other spermatid-specific transcript variants described [52]. In all cases, reactions were performed in triplicate on two independent sets of germ cells. Expression results were normalized to their corresponding *18S *rRNA content. For *Dnmt3L *expression analysis in male germ cells, fold changes in expression were determined in relation to the expression of that gene in E13.5 gonocytes for prenatal germ cells or in pachytene spermatocytes for postnatal germ cells; all other quantities are expressed as *n*-fold differences relative to the expression of that gene in these respective cells types. For the analysis conducted on *Dnmt3L *mutant germ cells, fold changes in expression were determined in relation to the expression in *Dnmt3L*^-/-^germ cells. Representative data for one set of germ cells are presented as mean ± SD.

### Histology

For histological examination, testes were immersed in Bouin's fixative (BDH Inc, Toronto, ON, Canada) for 4 hours, dehydrated, and embedded in paraffin. Sections (5 μm) were cut, mounted on glass slides, deparaffinized with xylene, and stained with hematoxylin and eosin. A Zeiss AxioImager Z1 microscope was used to view the slides and pictures were taken using a digital camera and the AxioVision 4.5 software (Carl Zeiss Canada Ltd, Toronto, ON).

### Germ Cell Counts

The paraffin-embedded testes used for histological analysis were cut into serial sections, with every fifth section used for germ cell quantification. The monoclonal germ-cell nuclear antigen 1 antibody GCNA1 was used to identify germ cells [39]. Briefly, rehydrated sections were incubated with undiluted primary antibody at 37°C for one hour, rinsed in PBS and incubated with a biotinylated anti-rat IgG secondary antibody (Vector Laboratories) for 30 minutes at room temperature; slides were then incubated with Vectastain Elite ABC reagent for one hour (Vector Laboratories), couterstained with Hematoxylin QS (Vector Laboratories) and mounted with Permount (Fisher Scientific, Fairlawn, NJ). Slides were viewed using a Zeiss AxioImager Z1 microscope (Carl Zeiss); germ cells were counted by an individual blinded to the slide identities and are reported per 2000 Sertoli cells as described [58]. Results are presented as means ± SEM.

### Immunofluorescence

Testes were fixed for four hours in Ste Marie's fixative as previously described [59] and were embedded in paraffin. Five-micrometer sections were rehydrated, blocked in 3% bovine serum albumin, 0.1% PBS-Tween 20 (blocking buffer) and incubated with the monoclonal mouse anti-phospho-histone-H2AX primary antibody diluted in blocking buffer for one hour at 37°C (1:500; Upstate Biotechnologies Inc., Charlottesville, VA). Rinsed sections were then incubated with a biotinylated anti-mouse IgG secondary antibody diluted in blocking buffer (1:500, Vector Laboratories) for 30 minutes at room temperature, followed by incubation with Avidin-AlexaFluor488 (Molecular Probes/Invitrogen) for one hour at room temperature in the dark. Slides were mounted with Vectashield containing DAPI (Vector Laboratories) and analyzed using a Zeiss AxioImager Z1 microscope (Carl Zeiss). Tubules containing at least one positive γ-H2AX cell, or positive tubules, were counted by an individual blinded to the slide identities and were reported as incidence of positive tubules per 100 tubules. Results are presented as means ± SEM.

### Restriction Landmark Genomic Scanning

Genomic DNA was isolated from whole testes of 10 dpp *Dnmt3L_+/+_, Dnmt3L_+/-_ and Dnmt3L_-/- _*mice, as well as sperm and liver from adult *Dnmt3L*_+/+ _mice using proteinase K followed by phenol extraction. Restriction landmark genomic scanning (RLGS) analysis was performed as described by Okazaki and colleagues [60]. Briefly, two-dimensional spot profiles were produced by digesting genomic DNA with the methylation-sensitive restriction enzyme NotI followed by radioactive end-labeling. RLGS gels were exposed to a phosphorimager screen (Kodak, Rochester, NY) and were analyzed using the ImageQuant v5.1 software from GE Healthcare (Piscataway, NJ). Densitometry values were obtained by comparing spot density values of a spot of interest to approximately 10-15 surrounding spots of unchanged intensity.

### Isolation of Dnmt3L mutant germ cells and DNA Methylation Analysis

*Dnmt3L*^+/- ^females were crossed with GOF18/deltaPE-Oct-4/GFP males to obtain [*Dnmt3L*^+/-^, GFP^+^] mice. Males and females with the proper genotype were crossed to obtain GFP^+ ^males with the three possible *Dnmt3L *genotypes; paired testes were collected at 6 dpp and were processed as mentioned above to allow for the isolation of *Dnmt3L *(+/+), (+/-) and (-/-) GFP^+ ^primitive type A spermatogonia by flow cytometry. RNA and DNA were simultaneously extracted using the AllPrep DNA/RNA Mini kit according to the manufacturer's protocol (Qiagen); the RNA was used in qRT-PCR analyses as described above, while DNA methylation analyses were carried out on the DNA. Quantitative analysis of DNA methylation using real-time PCR, or qAMP, was conducted to analyze the DNA methylation status of a number of sequences as described [44]. Briefly, the DNA was either mock digested (sham group), or digested with methylation-sensitive restriction enzymes (NotI, HpaII or HhaI), which cleave DNA if the restriction site(s) are unmethylated, or with a methylation-dependent restriction enzyme (McrBC), which cleaves DNA only if it is methylated. For a given sequence, primers were designed to flank the restriction sites of interest and real-time PCR was performed on the different digested templates using the QuantiTect ™ SYBR^® ^Green PCR kit (Qiagen) according to the manufacturer's suggested conditions for use of the Mx3000P PCR machine (Stratagene). Primer sequences for *U2af1-rs1 *are the same as in [44], primers for *H19, Dlk1-Gtl2 *and *Rasgrf1 *are from [51], and primers for the 31 sites analyzed on chromosome 4 and for the 30 sites analyzed along chromosome X are from [42] and are listed in Additional file 1. The cycle threshold (C_t_) values obtained for the different digested templates are expressed relative to the sham digested group; any differences in C_t _values (ΔC_t_) are indicative of differences in the methylation status of the given sequence of interest. Since each successive round of PCR amplification results in approximately a 2-fold increase in the amount of amplicon, a percentage methylation value can be extracted from the ΔC_t _values. Thus, a ΔC_t _of 1.0 is indicative of 50% template cleavage; 2.0, of 75% template cleavage, and so on. For methylation-sensitive restriction enzymes, the following formula can be used to described the relationship of ΔC_t _to percent methylation: %methylation = 100(2^-ΔCt)^); the relationship follows the inverse function for methylation-dependent restriction enzymes: %methylation = 100(1–2^(ΔCt)^). For the chromosomal analyses, the %methylation values obtained for each different enzymatic digest for a given loci were averaged to produce the percent value attributed to each position. Statistical analyses were conducted using SigmaStat v3.0 (SPSS). As data sets did not have equal variance, Mann-Whitney rank sum tests were ran; a P value of < 0.05 was considered to be significant.

## Abbreviations

DMR Differentially methylated region

DNMT DNA methyltransferase

Dpp Day postpartum

E Embryonic day

GFP Green fluorescent protein

PGC Primordial germ cell

qAMP Quantitative analysis of DNA methylation using real-time PCR

qRT-PCR Real-time (quantitative) RT-PCR

RLGS Restriction landmark genomic scanning

## Competing interests

The author(s) declares that there are no competing interests. 

## Authors' contributions

SL was responsible for *Dnmt3L *and *GOF18/deltaPE-Oct4/GFP *mice husbandry and genotyping, all germ cell isolations and tissue collections, in addition to performing all qRT-PCR and immunofluorescence studies and part of histological analyses; she also participated in assay and primer design for qAMP. CCO carried out RLGS and all qAMP experiments. ORN performed part of histological analyses and all germ cell counts. DB and THB provided the *Dnmt3L *mice. SL and JMT were responsible for the study design and manuscript drafting. CCO, ORN, DB and THB revised the manuscript and all authors read and approved the final manuscript.

## Supplementary Material

Additional File 1 qAMP primer sequences used to determine DNA methylation levels of non-CpG-island sequences on chromosomes 4 and X in *Dnmt3L*^-/- ^germ cells. Click here for file
