# Supplementary material for: Loss of spermatogonia and wide-spread DNA methylation defects in newborn male mice deficient in DNMT3L
Source: BMC Dev Biol. 2007 Sep 18;7:104. doi: 10.1186/1471-213X-7-104 (PMC2212652; doi:10.1186/1471-213X-7-104)
Supplement: Additional File 1 — qAMP primer sequences used to determine DNA methylation levels of non-CpG-island sequences on chromosomes 4 and X in Dnmt3L-/- germ cells. [file 1471-213X-7-104-S1.doc]

**Table 1:** qAMP primer sequences used to determine DNA methylation levels of non-CpG-island sequences on chromosomes 4 and X in *Dnmt3L^-/-^* germ cells.

| **Position (mm 7)** | **Forward primer (5'-3')** | | **Reverse primer (5'-3')** |
| --- | --- | --- | --- |
| **Chromosome 4** | | | |
| 4872334-4872813 | ACCCTTCAAAACCCGTGAAT | | GCCTGAATCTTGCTCTTTGCAT |
| 9927753-9928179 | GAAAGGGGAACAGGGGAGTA | | GGCACCTAGCATCTTGGAGA |
| 15477713-15478091 | GTGTTGGCTAATGAGGAGGA | | GAAGGAGAAAGGATGCTGGA |
| 19850460-19850826 | CTATGACTCCCCACGTCACA | | GTCATGCGGAAGACATCTGA |
| 24806042-24806420 | GGAAACAGAGCTCTCTGGAA | | ACAGCTAACCCAATGGCTCT |
| 30339739-30340307 | TTGAACAGCATGCCTCTCT | | TTTAACTGCGCTGTGGAGAA |
| 35145276-35146275 | TCATCAAGGGCAGAGGAAAT | | TTTCGAGAAGGACGGAGGT |
| 40155437-40155738 | ACCACACAGACCTCCTCTCA | | CTCAAAGCAGCCACGACTGT |
| 45282104-45282572 | TCATCAGTGACCCCTCTTCC | | CTGGACCAGCTCTTCCTCAT |
| 50216637-50217105 | TGGCTAGGGAAGAGGTGAGA | | CTTCTTCCCTTGTGGCTTGA |
| 55202844-55203145 | TTTGAGAGAAGGCAGCATGA | | AAGGCCTTCGTCGTTAGACA |
| 59969750-59970164 | GTGCCACATGGTGTGGTAAA | | ATATGCCGTATTGCACAACC |
| 65403458-65403793 | CCCAGGGTAAAAAGGATCA | | AATCGTCTCGAACTCGCTCA |
| 70217560-70217910 | GGGGCTTTAAATGGGAAACA | | TCAAGCAGGAAGAGCTGGATA |
| 75238764-75239513 | CCCAGATACCAAGGTGTGTCT | | GGCTGACAGGTGAACTGAGA |
| 80266828-80267395 | CATGTGTCCCCGTTTCTTGT | | CAGCTTGGTCACAACCATCA |
| 85800348-85800847 | CACCCCATCTCCCATTTCTA | | AGGATCACCACGAAACAGGT |
| 90473664-90474084 | GGACAAGGGGGCTTTCTTTCT | | GGGAATGGAGCTGTATGGT |
| 96111617-96112110 | AGCTTCCCACTTTCCAACAA | | GCCTTTCAGCTACAGTTCCAA |
| 100277854-100278187 | AAAACCAACAGGCCTGAGAA | | TCGTCGTCAAAAAGGTCAGA |
| 105596524-105596856 | AGCAACAGCAGCAACTGAAC | | TCCCTGGTTGATCCTGTGTA |
| 110703297-110703796 | GAGAGGAACCTGAGGCTTGA | | CAGCAGAGACGGGAGACAAT |
| 115472605-115473015 | TTCATGGCATCCCTACCAGT | | TCTTGCTGTGACTGCATCCT |
| 120466404-120466860 | CTGGAAGAACATGGCAAGTGA | | TCCTCCCTCTGTTCTCTGGT |
| 125407419-125407757 | ACCATGGAAAAGGAGCAACA | | GCCAGGTCTGGATACAAGGA |
| 129975322-129975744 | TCCACTGTGTTCAGAAGCAA | | GGAATAACCGGTCATCCAAA |
| 135498472-135498890 | CCCGAGGTCATGAGAAAGAA | | CTGTTTCCTGGGGTTGTGAT |
| 140499164-140499582 | TGCACTGGAACAGGACTGAG | | GAGGGGATGTAAACGGGAGT |
| 144580754-144581253 | TCTGCTAGCTCCTCCTGCTT | | GGAGGTGTTGTGGCTAGCTT |
| 150486904-150487236 | CGAACGGAAGAAAAGGACTG | | TGTAGTGTCTGGGAGGGACA |
| 155169477-155169776 | AATGTGAGGGTCGTTGCACT | | TGTAGGCCAGCTTCCTTCAT |
| **Chromosome X** | | | |
| 4890124-4890623 | GGCAGTAAGGGGACAGGTTA | CCTTGACAGACCAGCTCTGAA | |
| 9827238-9827737 | AGCGCTGACTGGAAGTCATT | CTCCTCCCTCACCTTTTGAA | |
| 15319447-15319946 | ACCGGTAAGTGGCAGTAAGT | TTCTCCAGATGGTGCTCCT | |
| 20292767-20293099 | AGTAATAGACGGGGCCAGGA | CTGCTTTGGGAGAAGTGGTCT | |
| 35736464-35736818 | TTTCCAGCGCCAATAGTTGT | TGTGGTGATCACATCACTTCAG | |
| 40656582-40656936 | CTGGCATCACTGCATCTATGA | CAGAGTGGTTGTGCTCCTGAT | |
| 45749431-45750930 | AAGAATCTAGGGCCCTCTTTG | GTGGACCGAGACAAAGGAAA | |
| 48789755-48790109 | TCCCCAGGAACCTTCTACCT | GTGTAGGAGGATGGCAGGAA | |
| 56195598-56196098 | CAGACTCCAGCAACAAAGGA | CCCTTGAGAGCAAATCGAAG | |
| 61330784-61331547 | AGCACGGGCTGCTACAAA | CACTGCCTCTTCTGTGTCCA | |
| 66502472-66502918 | AATTGCCCAACTGCATACAA | AATGCTGGGCTTCACCAATA | |
| 71408206-71408702 | TAGCTAGCGGGGTATGACTT | CCTTGAAGGAGAACAGCATA | |
| 76236203-76236748 | TGCTCACTGTGCTACAACCA | GTCCATGAGCCTCTCCCTTA | |
| 80709428-80709859 | AGTCCAGGAGCAACTGGCTA | CGAGGCAGCTCAAAGGTAGA | |
| 85404289-85404720 | ATTGGGTTCATCTGGCTACA | CCTGATTATCACAAGCATTCATC | |
| 90325997-90326375 | CTTTTGCCAACGGTCCTCTA | CCCGAGGAAAATGAACCTGT | |
| 95723738-95724169 | TGTGTCCCGAGCATTGATTA | GGGCTGAGCAAACCTTTCTT | |
| 100788840-100789271 | GACCCACATGATCAAAGTGCT | AAGAAAATCCTGGCTGCTGA | |
| 105645103-105645481 | TCAGAGCCATGGAATGTGTT | TTTGGAGCTCATTCAGACCT | |
| 110765173-110765604 | GACAAATGCTATCTTTAATGGCTGT | TTGAGAGAAACGCACCAAAA | |
| 115633810-115634309 | AGGATTTCGTCCAATCTCA | TGTATGAAATGCACTGTCTTGT | |
| 121249040-121249469 | TGAAGGTGGAAAAATCTGCTGT | TGCATGTGATACTTACTCCCTATGA | |
| 126210018-126210447 | CTTCCTTCTCTTTTGCCTTTGA | GCAGAATTACTGGGAGAGAAAT | |
| 130693473-130695722 | GGCTCCTTTGCTCATCTTCT | GGGTGCCTGTGTCTGATTCT | |
| 136254525-136254954 | GCCTTAGAAGCCATTATAGTTTTGTC | TCTCTGAACCCTTTGAAAGCA | |
| 141149803-141150371 | CCAGCTGCTTGATTGCTTCT | GCTTTTAAAGGAGGGGCTTC | |
| 146231833-146232219 | TCCTAAATACTCTTGGGGTTTGG | TCAGCAATAAAAGGGGAATAG | |
| 151368505-151369254 | CCTTCAGAGACTTTCTCCCTTT | GGCAGTCTGCTAGAATGTGCT | |
| 156408103-156408651 | CAGCCTTGCTGATCTGACAT | TTGTGTCACTGCCTCCATAA | |
| 161246279-161246665 | AGCATGAGCCCACTCTTGA | TGGACAGGTGTCACTTGCAT | |
